# Quality improvement in pre-hospital critical care: increased value through research and publication

**DOI:** 10.1186/1757-7241-22-34

**Published:** 2014-05-29

**Authors:** Marius Rehn, Andreas J Krüger

**Affiliations:** 1 Department of Research, Norwegian Air Ambulance Foundation, Drøbak, Norway; 2 Department of Health Studies, Faculty of Social Sciences, University of Stavanger, Stavanger, Norway; 3 Department of Anaesthesia and Intensive Care, Akershus University Hospital, Lørenskog, Norway; 4 Department of Anaesthesia and Intensive Care, St. Olavs Hospital, Trondheim, Norway

## Abstract

Pre-hospital critical care is considered to be a complex intervention with a weak evidence base. In quality improvement literature, the value equation has been used to depict the inevitable relationship between resources expenditure and quality. Increased value of pre-hospital critical care involves moving a system from quality assurance to quality improvement. Agreed quality indicators can be integrated in existing quality improvement and complex intervention methodology. A QI system for pre-hospital critical care includes leadership involvement, multi-disciplinary buy-in, data collection infrastructure and long-term commitment. Further, integrating process control with governance systems allows evidence-based change of practice and publishing of results.

## Complex interventions in pre-hospital critical care

Emergency Medical Service (EMS) personnel aim to provide a seamless continuation of the critical care environment from the scene of injury or onset of acute illness until definitive treatment. To avoid that geography determine patient care, EMS providers must overcome austere pre-hospital conditions as well as geographical and logistical challenges. The evidence-base for this practice remains weak and research is generally underfunded [1,2]. The typical critical care pathway involves care provided by numerous providers throughout a chain of coupled environments. Unsurprisingly, a typical critical care patient is reported to experience an average of 178 clinical events per day [3]. This yields a high number of possible outcomes and interconnected initiatives that suggests that pre-hospital critical care is a complex intervention. Research into complex interventions often requires application of special study designs [4,5].

### Quality assurance versus quality improvement

**Figure 1:**
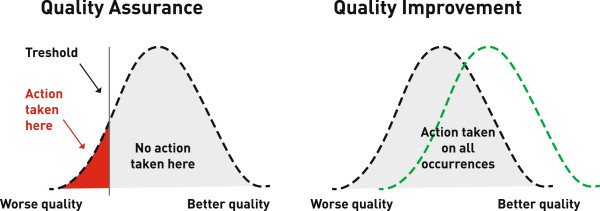
**Quality assurance versus quality improvement.** Source: Institute for healthcare improvement; Dr. Scoville, Dr. Lloyd (permission for reprint granted).

Traditionally, quality of care has been assured through initiatives directed towards outliers at the poorest spectrum of practice. This left those performing above the arbitrarily chosen quality threshold without any formal improvement initiatives. The growing understanding that all spectrums of care can improve, has led to Quality Improvement (QI) systems that has the potential for parallel shifts of entire services towards better quality (c.f. Figure 1 for Quality Assurance versus Quality Improvement). In time- and monetary constrained environment, EMS providers have integrated QI elements such as morbidity and mortality reviews and clinical audit into daily practice [6,7]. These initiatives serve as the foundation for a QI system, but results are rarely published and methods are often unrefined. However, tools to mature these systems exist in the QI literature [8-10]. The relevance of such systems is exemplified with the World Health Organization Guidelines for trauma QI programme [11]. This programme enables health care institutions to better monitor care services, detect areas of potential improvement to more effectively enact and launch initiatives to improve the quality of care.

### Quality improvement integrated in pre-hospital critical care

A major challenge in QI lies in identification of optimal quality indicators [12,13], and how to capture these indicators in the operational context of pre-hospital critical care [14]. A recent thesis emphasise the need for novel thinking and subsequent redesign of documentation systems for pre-hospital critical care [14]. The thesis also argues for development of systems that integrate multiple data sources and automatically include hospital-based outcome measures into activity databases. Based on the concept of complex interventions, finding the true quality of pre-hospital critical care might not solely be based on outcome measures such as mortality. Long-term hard outcome measures are heavily influenced by the quality delivered throughout the complete episodes of critical care [15,16]. As such, efforts must be made to develop patient-centred quality dimensions for the specific pre-hospital critical care interval. Designing data collection tools to map risk-adjustment measures, therapeutic-, and hard outcomes for each phase of the episode of critical care might be the way forward [17].

The QI journey starts when you start to measure and statistical process control is a suggested method that allow better comprehension and communication of data from QI efforts [18]. Such data should be integrated in governance systems so EMS providers can close the loop by adjusting practice.

**Figure 2:**
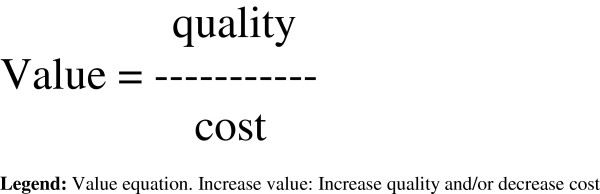
**Value equation.** Increase value: Increase quality and/or decrease cost.

The value equation has been much used to depict the inevitable relationship between resource expenditure and quality [19]. By increasing the numerator (quality) while maintaining the denominator (cost), the value will increase (c.f. Figure 2 for value equation) [20]*.* In the context of multiple possible QI initiatives, decision-makers may rank projects according to expected value yield.

### Dissemination of quality improvement research

Writing is a significant part of your work to improve the quality and cost effectiveness of your critical care practice. However, papers depicting QI initiatives have been relatively few, and reporting has been insufficient. Accordingly, Standards for QUality Improvement Reporting Excellence (SQUIRE) were developed to facilitate more and improved reporting of QI studies [21]. Several journals have formally adopted them as editorial policy, including the Scandinavian Journal of Trauma, Resuscitation and Emergency Medicine [22].

The SQUIRE guidelines provide EMS personnel with a framework that clarify hypothesis, verify observations and warrants inferences. Further, it may speed up the dissemination of important innovations [23]. Lastly, it formalize QI initiatives as research, making it more relevant for Research Ethics Committees to grant formal approval often required by journal Editors.

EMS providers have two duties when they attend their shifts: 1) Do their job and 2) Do it better. Increased value of pre-hospital critical care carries moving from a system of Quality Assurance to Quality Improvement. Further, EMS providers should gain consensus on quality indicators to be integrated in existing QI and complex intervention methodology.

They should aim to implement QI systems that include leadership involvement, multi-disciplinary buy-in, data collection infrastructure and long-term commitment. Further, they should merge process control with governance systems and methodically publish results. By integrating QI to our daily pre-hospital critical care, we may increase the value of our efforts and ultimately prevent that patients die before they are done living.

## Abbreviations

EMS: Emergency medical services; QI: Quality improvement; SQUIRE: Standards for quality improvement reporting excellence.
